# Supplementary material for: Structural insight into the allosteric inhibition of human sodium-calcium exchanger NCX1 by XIP and SEA0400
Source: EMBO J. 2023 Dec 15;43(1):14–31. doi: 10.1038/s44318-023-00013-0 (PMC10897212; doi:10.1038/s44318-023-00013-0)
Supplement: Supplementary file 1 — Appendix [file 44318_2023_13_MOESM1_ESM.pdf]

Appendix

Mechanistic insight into allosteric inhibition of human sodium-calcium exchanger NCX1 by XIP and SEA0400

Yanli Dong, Zhuoya Yu, Yue Li, Bo Huang, Qinru Bai, Yiwei Gao, Qihao Chen, Na Li, Lingli He, Yan Zhao*

* Correspondence emails: zhaoy@ibp.ac.cn (Y.Z.)

Table of contents

Appendix Figures

Appendix Figure S1: Functional characterization and purification of NCX1.3…………………..3

Appendix Figure S2: Cryo-EM data processing of NCX1.3 with inhibitor SEA0400……………4

Appendix Figure S3: Full-length sequence alignment of NCX homologous……………………6

Appendix Figure S4: Molecular dynamic simulation analysis of binding poses of SEA0400….7

Appendix Tables

Appendix Table S1: Primers used in this study………………………………………………….8

Appendix Table S2: Cryo-EM data collection, refinement, and validation statistics……………9

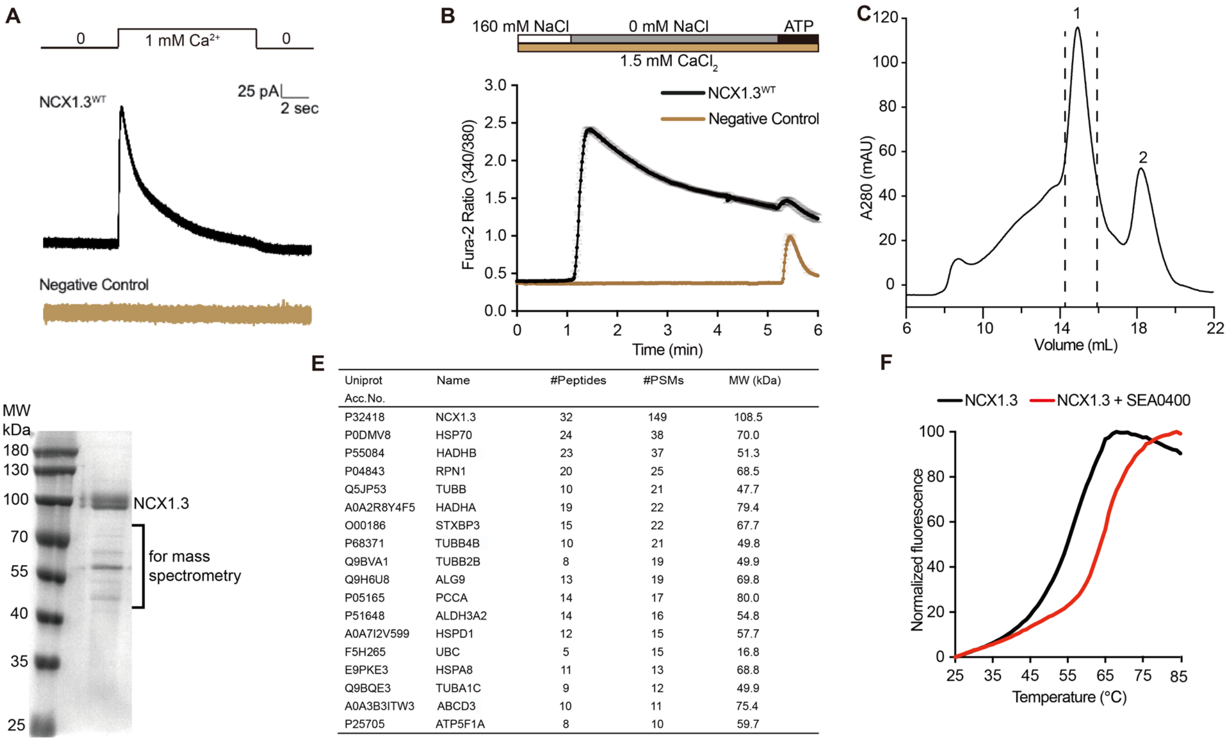

Uniprot Acc.No.	Name	#Peptides	#PSMs	MW (kDa)
P32418	NCX1.3	32	149	108.5
P0DMV8	HSP70	24	38	70.0
P55084	HADHB	23	37	51.3
P04843	RPN1	20	25	68.5
Q5JP53	TUBB	10	21	47.7
A0A2R8Y4F5	HADHA	19	22	79.4
O00186	STXBP3	15	22	67.7
P68371	TUBB4B	10	21	49.8
Q9BVA1	TUBB2B	8	19	49.9
Q9H6U8	ALG9	13	19	69.8
P05165	PCCA	14	17	80.0
P51648	ALDH3A2	14	16	54.8
A0A7I2V599	HSPD1	12	15	57.7
F5H265	UBC	5	15	16.8
E9PKE3	HSPA8	11	13	68.8
Q9BQE3	TUBA1C	9	12	49.9
A0A3B3ITW3	ABCD3	10	11	75.4
P25705	ATP5F1A	8	10	59.7

Appendix Figure S1. Functional characterization and purification of NCX1.3. Related to Figure 1, Figure 3, and Figure 4.

A Representative Na^{+}_{i} dependence of outward Na^{+}-Ca^{2+} exchange current traces in HEK293T cells transfected with $NCX1.3^{WT}$ and untransfected as negative control by whole-cell patch clamp. Outward currents were activated by changing the extracellular Ca^{2+} from 0 to 1 mM for 10s.

B Representative traces of cytosolic Ca^{2+} measurements using Fura-2 AM as an indicator in HEK293T cells transfected with $NCX1.3^{WT}$ and untransfected as negative control. The change of solution was marked by time breaks. The Krebs' buffer including 160 mM NaCl was changed Na^{+}-free $NMDG^{+}$ buffer (0 mM NaCl) at one minute. Subsequently, 500 μM ATP was added at the end as a control for cell viability. Data represent the mean ± SEM of recordings. The Fura2 ratio (340/380) was used as a quantitative indicator of intracellular $[Ca^{2+}]$.

C Size-exclusion chromatogram (Superose 6 increase) of the purified protein sample of NCX1.3. The peak 1 (marked within black dashed lines) was pooled and concentrated for cryo-EM study. The peak 2 represents cleaved mCherry.

D Coomassie-blue-stained SDS-PAGE gel of the NCX 1.3. The protein band is labeled. The experiments were repeated independently more than 3 times with similar results.

E The results of mass spectrometry analysis for NCX1.3, which only displayed a PSM (Peptide-Spectrum Match) score greater than 10.

F Thermal stability assay as described in the methods section. The addition of 100 μM SEA0400 increased the thermal stability of NCX1.3. The experiments were repeated independently 4 times, consistently yielding similar results.

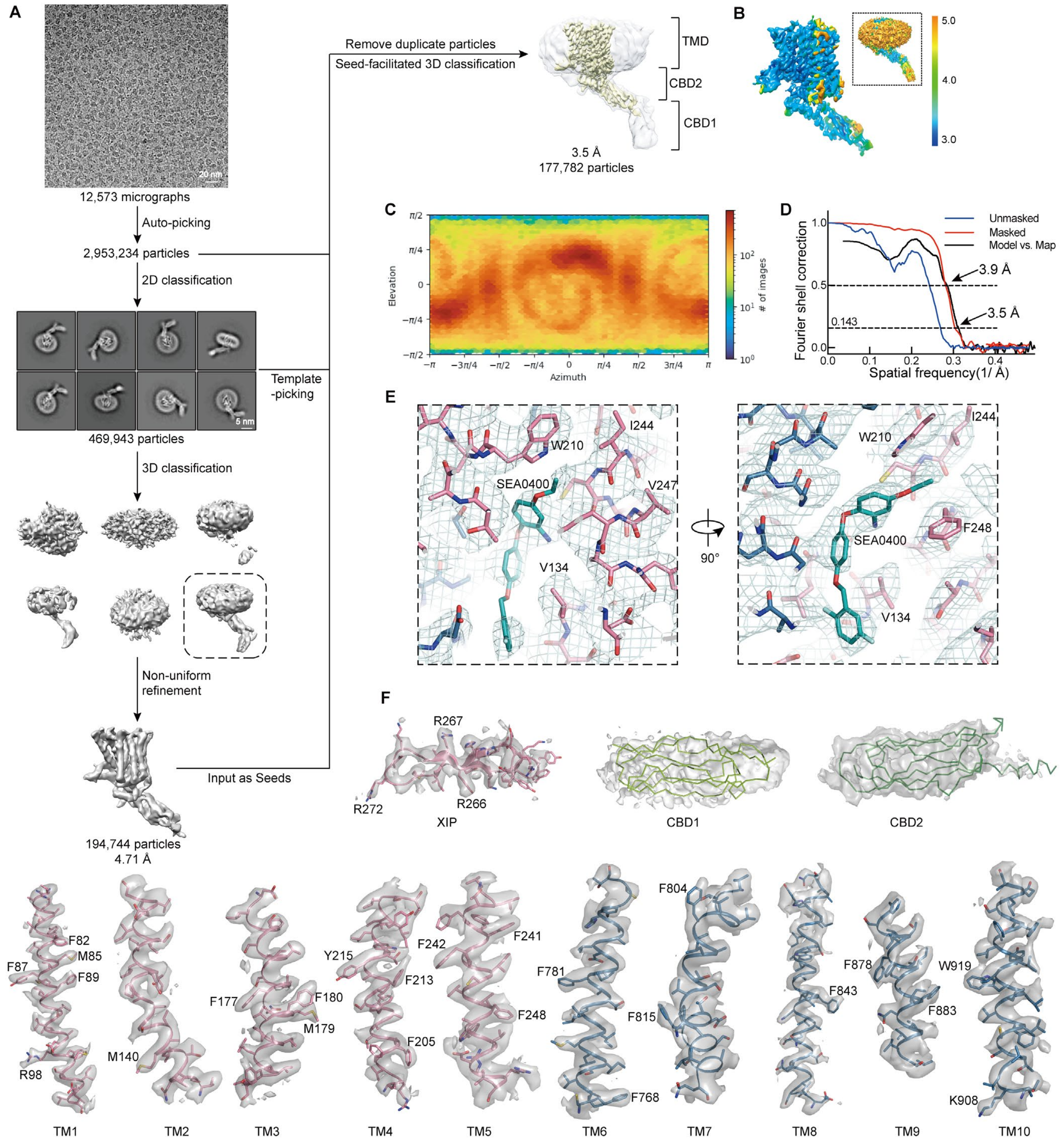

Appendix Figure S2. Cryo-EM data processing of NCX1.3 with inhibitor SEA0400. Related to Figure 1.

A Flow chart of cryo-EM data processing. A representative motion-corrected cryo-EM micrograph (bar = 20 nm) of a total of 12,573 movie stacks and 2D class averages of distinct secondary structure features from different views of NCX1.3^{SEA0400} are shown, respectively. Particles were picked in cryoSPARC, and were used to three rounds of 2D classification. Only classes featuring transmembrane helices and discernible soluble domain were selected and subjected to 3D classification. One of six classes showing a discernable shape of NCX1.3 was set as the good reference map for the subsequent heterogeneous refinements. The resulting map with resolution of 3.5 Å displayed in yellow surface (threshold: 0.10) was

overlaid with its grey surface (threshold: 0.41). TMD, CBD1 and CBD2 were labeled. Details of data processing can be found in Materials and Methods.

B Local resolution map of NCX1.3$^{\mathrm{SEA0400}}$.

C The angular distribution of the final reconstruction.

D Fourier Shell Correlations (FSC) of the final map of NCX1.3$^{\mathrm{SEA0400}}$, calculated between two independently refined half-maps before (blue) and after (red) post-processing. The FSC curve calculated between the cryo-EM density map and the structural model are shown in black.

E SEA0400 (sticks with carbon atoms in salmon) binding pocket in detail, with tiagabine density in mesh (contoured at 4.8σ) and interacting residues in stick representation.

F The cryo-EM density map and atomic model of TMs, XIP and CBDs of NCX1.3$^{\mathrm{SEA0400}}$. The cryo-EM maps are shown as grey surface.

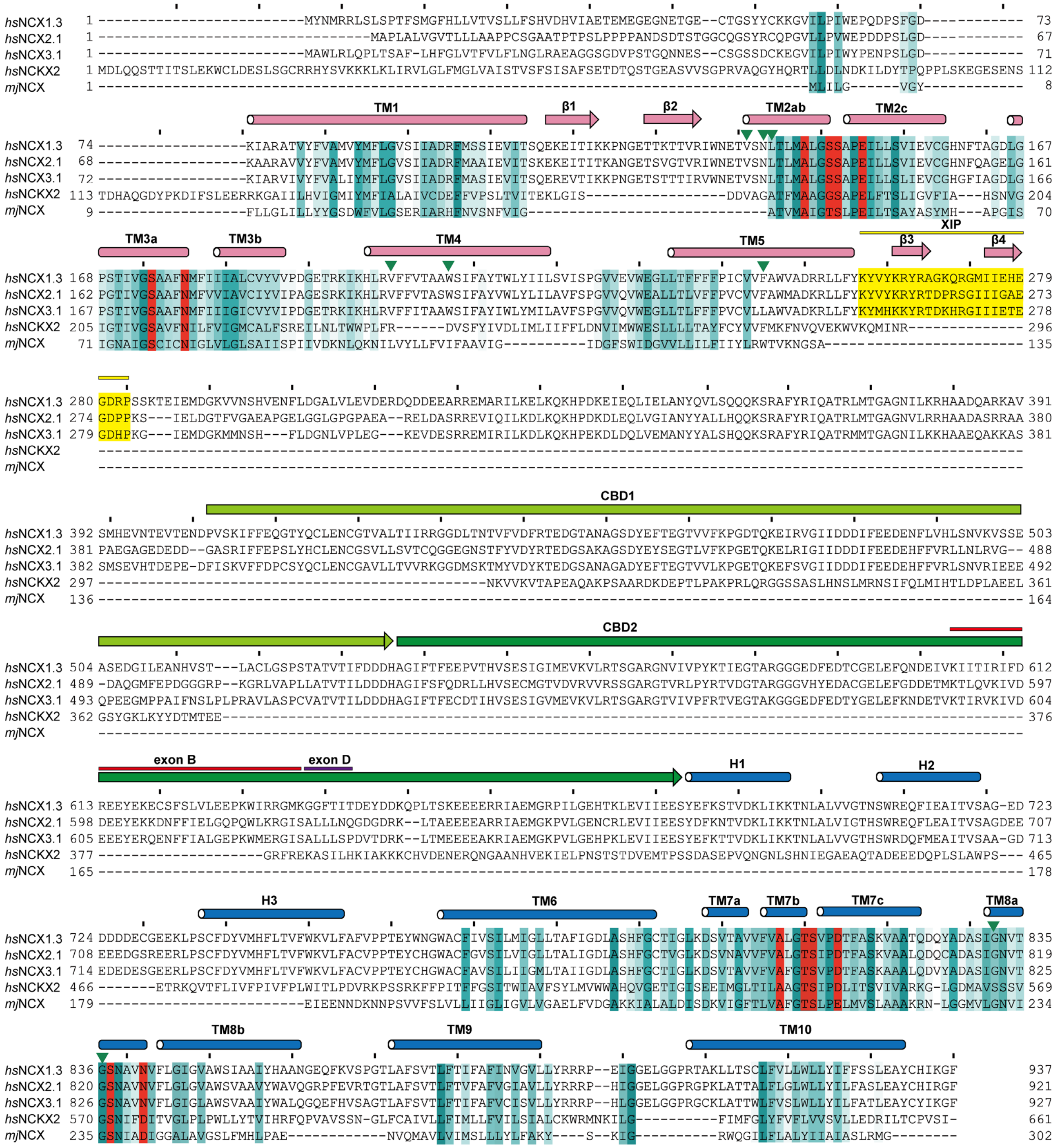

Appendix Figure S3. Full-length sequence alignment of NCX homologous. Related to Figure 2, Figure 3, and Figure 4.

The sequence alignment among human NCX1.3, human NCX2.1, human NCX3.1, human NCKX2 and *Methanococcus jannaschii* NCX. Secondary structural elements of hsNCX1.3 are indicated and marked above the sequence alignment. The XIP region is shaded in yellow. The exon B and exon D of NCX1.3 CBD2 are marked as red lines and purple lines, respectively. Na^+/Ca^{2+} binding sites are highlighted in red. Residues important for SEA0400 binding are marked as green triangles.

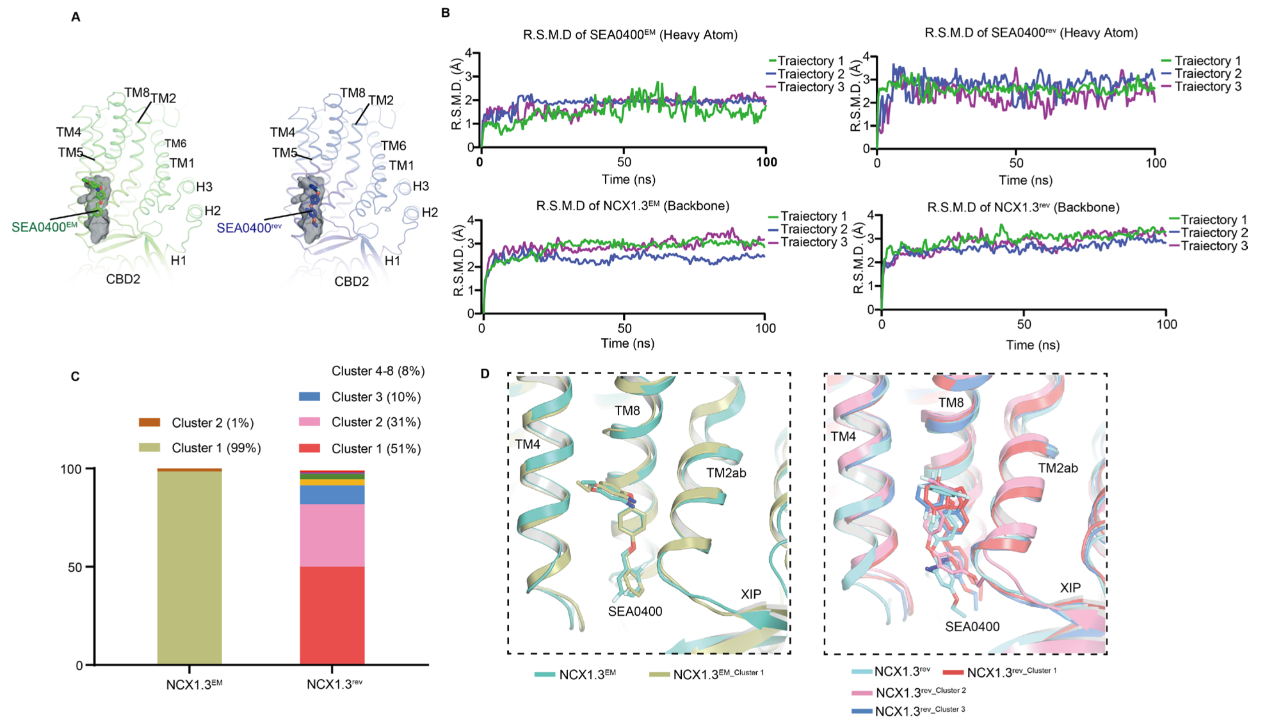

Appendix Figure S4. Molecular dynamic simulation analysis of binding poses of SEA0400. Related to Figure 4.

A Initial structures used in MD simulations. Two sets of simulations, referred to as the NCX1.3^{EM} and NCX1.3^{rev}, were included in the MD study with SEA0400 initially placed in the pocket in two distinct orientations. For NCX1.3^{EM}, SEA0400 adopts the same poses as observed in the cryo-EM structure (SEA0400^{EM}). On the other hand, in the NCX1.3^{rev}, SEA0400 assumed a reversed pose (SEA0400^{rev}).

B R.M.S.D. plots for ligand and protein backbone during MD simulation. R.M.S.D. plots were generated for both SEA0400 and the protein backbone. The calculation of backbone R.M.S.D. for NCX1.3 using residues 51-283, 683-720, and 736-937, with the initial structure serving as the reference. The calculation of SEA0400 R.M.S.D. involved the heavy atoms of SEA0400, using its initial structure as the reference. Results from three independent trajectories were distinguished by different colors.

C Cluster populations obtained from clustering analysis. The clustering analysis was conducted respectively for NCX1.3^{EM} and NCX1.3^{rev}, each involving three independent 100 ns trajectories. Heavy atoms from SEA0400 and the pocket (protein residues within 5 Å of SEA0400) were employed in the clustering analysis, with a 1.5 Å R.M.S.D. cut-off.

Structural comparisons of initial poses of SEA0400 with representative poses derived from clustering analysis. The left panel demonstrates the high consistency between the cryo-EM structure and the representative structure from the NCX1.3^{EM}, indicating the high stability of SEA0400 binding in the observed cryo-EM pose. Conversely, the right panel depicts diverse poses observed in the NCX1.3^{rev}, suggesting the low stability of such a binding pose. The NCX1.3 model is shown in cartoon and **Appendix Tables**

Appendix Table S1. Primers used in this study

Name	Sequence
NCX1.3-F	ATGTACAACATGCGGCGATTAAGTC
NCX1.3-R	TCAGAAGCCTTTTATGTGGCAGTAG
NCX1.3$^{\triangle XIP}$-F	TGTTTTACAAGTATGTCTACGACAGGCCATCTT
NCX1.3$^{\triangle XIP}$-R	GTCTTAGAAGATGGCCTGTCGTAGACATACTTGT
NCX1.3^{IIQQ}-F	GCAGAGGGGGATGCACCACGAACATGAAGGAGACA
NCX1.3^{IIQQ}-R	TGTCTCCTTCATGTTCGTGGTGCATCCCCCTCTGC
NCX1.3^{C245F}-F	ATCTGTGTTGTGCTCGCTTGGGTAGCGGAT
NCX1.3^{C245F}-F	CAAGCGAACACAACAAGATGGGAAAGA
NCX1.3^{F248L}-F	ATCTGTGTTGTGCTCGCTTGGGTAGCGGAT
NCX1.3^{F248L}-R	ATCTGTGTTGTGCTCGCTTGGGTAGCGGAT
NCX1.3^{G832N}-F	TCCATAAATAACGTCACGGGCAG
NCX1.3^{G832N}-R	ACGTTATTTATGGAGGCGTCTGC
NCX1.3^{G836N}-F	AGGTAACGTCACGAACAGCAACGCGGTGAATGTCTTCCT
NCX1.3^{G836N}-R	AGGAAGACATTCACCGCGTTGCTGTTCGTGACGTTACCT
NCX1.3^{IWPP}-F	ACAACTGTGAGGCCTCCAAATGAAACAGTTTCTAAC
NCX1.3^{IWPP}-R	AACTGTTTCATTTGGAGGCCTCACAGTTGTCTTGGT
NCX1.3$^{\triangle \beta1\beta2}$-F	AGTCATCACATCTCAAATCTGGAATGAAACAGTTTCTAAC
NCX1.3$^{\triangle \beta1\beta2}$-R	CTGTTTCATTCCAGATTTGAGATGTGATGACTTCTATAGAGG

Appendix Table S2. Cryo-EM data collection, refinement and validation statistics.

	NCX1.3<br>(EMDB-36465)<br>(PDB 8JP0)
**Data collection and processing**	
Magnification	165,000 ×
Voltage (kV)	300
Electron exposure (e–/Å^2)	60
Defocus range (μm)	−1.2 ~ −2.2
Pixel size (Å)	0.82
Symmetry imposed	C1
Initial particle images (no.)	2,953,234
Final particle images (no.)	194,744
Map resolution (Å)	3.5
FSC threshold	0.143
Map resolution range (Å)	3.0 ~ 5.0
**Refinement**	
Initial model used (PDB code)	3V5S, 2DPK, 2KLT
Model resolution (Å)	3.5
FSC threshold	0.143
Model resolution range (Å)	3.3 ~ 3.9
Map sharpening *B* factor (Å^2)	148.2
Model composition	
Non-hydrogen atoms	5671
Protein residues	719
Ligands	46 (SEA0400)
B factors (Å^2)	
Protein	101.21
Ligand	59.16
R.m.s. deviations	
Bond lengths (Å)	0.004
Bond angles (°)	0.759
Validation	
MolProbity score	2.18
Clashscore	12
Poor rotamers (%)	0.30
Ramachandran plot	
Favored (%)	91.26
Allowed (%)	8.74
Disallowed (%)	0.00
