# Supplementary material for: Structural insight into the allosteric inhibition of human sodium-calcium exchanger NCX1 by XIP and SEA0400
Source: EMBO J. 2023 Dec 15;43(1):14–31. doi: 10.1038/s44318-023-00013-0 (PMC10897212; doi:10.1038/s44318-023-00013-0)
Supplement: Supplementary file 3 — Movie EV1 [file 44318_2023_13_MOESM3_ESM.zip › Movie EV1/Movie EV1 legend.docx]

Movie EV1 legend:

The morph between the experimentally determined inward open state structure NCX1.3^IF^ and the outward open state model NCX1.3^OF^*. The N-domain and C-domain of NCX (Sodium-Calcium Exchanger) are represented by red and blue cartoons, respectively, while the ion binding sites are shown as sticks. XIP (Exchanger Inhibitory Peptide) is depicted in yellow cartoon, and SEA0400 is represented by green sticks. SEA0400 and XIP lock NCX1.3 in the inward-facing conformation. Upon dissociation of SEA0400 and XIP from their respective positions, TMs1/6 of NCX1.3 undergo vertical sliding along the membrane plane, accompanied by conformational changes in TM2a/TM7a. As a result, the ion binding sites alternate exposure between the intracellular and extracellular sides, allowing for conformational switching between inward-facing and outward-facing states.
