# Supplementary figures and images for: Structural insight into the allosteric inhibition of human sodium-calcium exchanger NCX1 by XIP and SEA0400
Source: EMBO J. 2023 Dec 15;43(1):14–31. doi: 10.1038/s44318-023-00013-0 (PMC10897212; doi:10.1038/s44318-023-00013-0)

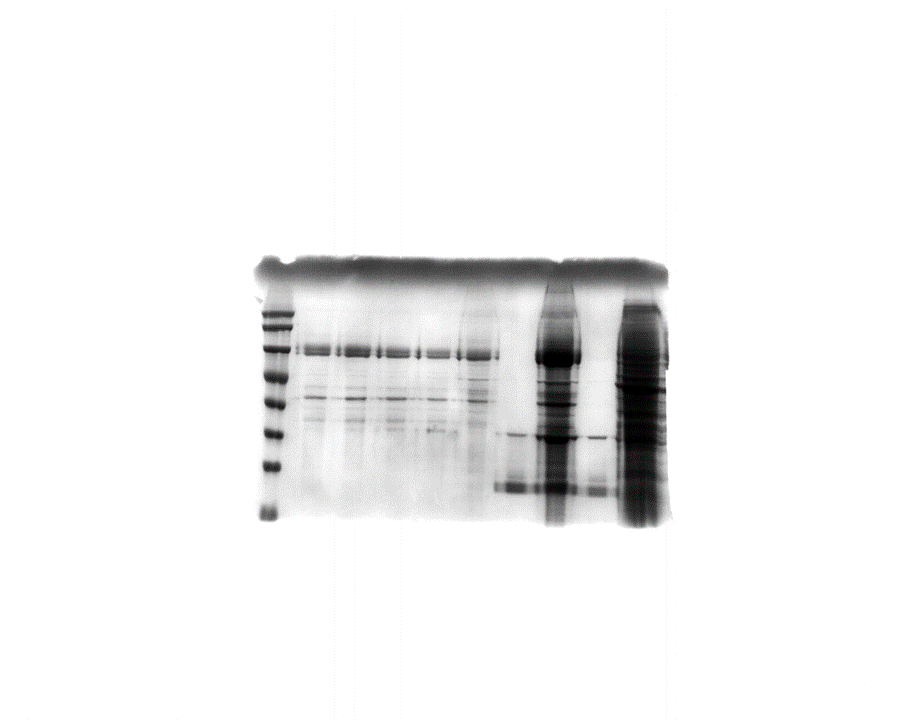

Supplement: Supplementary file 6 — EV and Appendix Figure Source Data [file 44318_2023_13_MOESM6_ESM.zip › Figure EV and appendix/SDS-PAGE gel.png]
