# Supplementary material for: Structural insight into the allosteric inhibition of human sodium-calcium exchanger NCX1 by XIP and SEA0400
Source: EMBO J. 2023 Dec 15;43(1):14–31. doi: 10.1038/s44318-023-00013-0 (PMC10897212; doi:10.1038/s44318-023-00013-0)
Supplement: Supplementary file 7 — Expanded View Figures [file 44318_2023_13_MOESM7_ESM.pdf]

Expanded View Figures

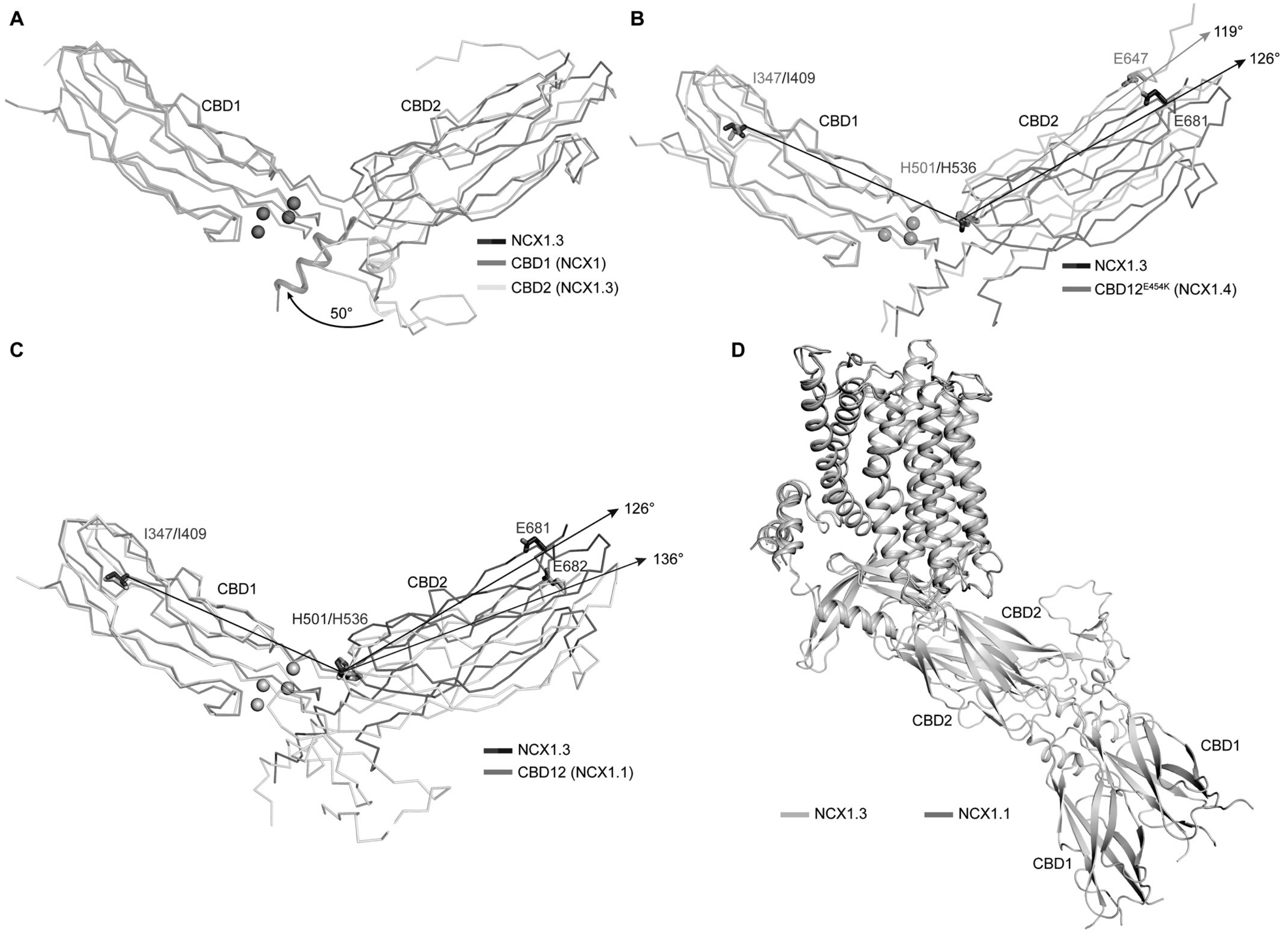

Figure EV1. Structural alignment of CBDs among human NCX1 isoforms. Related to Fig. 1.

(**A**) Structural comparison of CBDs between NCX1.3 and NCX1. The Ca^{2+}-bound CBD1 structure of NCX1 (PDB ID: 2DPK) is colored in yellow and the bound Ca^{2+} ions are displayed as yellow spheres. CBD1 and CBD2 of NCX1.3 in our structure are colored in green and dark green, respectively. The CBD2 of NCX1.3 (PDB ID: 2KLT) is colored in pink. (**B**) Structural comparison of CBDs between NCX1.3 and NCX1.4 ($CBD12^{E454K}$, PDB ID: 3US9) using the invariant CBD1 domains. CBD1 and CBD2 of NCX1.4 are colored in purple. The key residues are shown as sticks. The bound Ca^{2+} ions of CBD1 for $NCX1.4^{E454K}$ are displayed as purple spheres. The angles between CBD1 and CBD2 of NCX1.3 and NCX1.4 are marked, respectively. (**C**) Structural comparison of CBDs between NCX1.3 and NCX1.1 (PDB ID: 8SGJ). CBD1 and CBD2 of NCX1.3 in our structure are colored in green and dark green, respectively. (**D**) Structure superposition of NCX1.3 and NCX1.1 (PDB ID: 8SGJ) using TMD domain. NCX1.3 and NCX1.1 are shown as salmon and gray cartoon, respectively.

 © The Author(s)

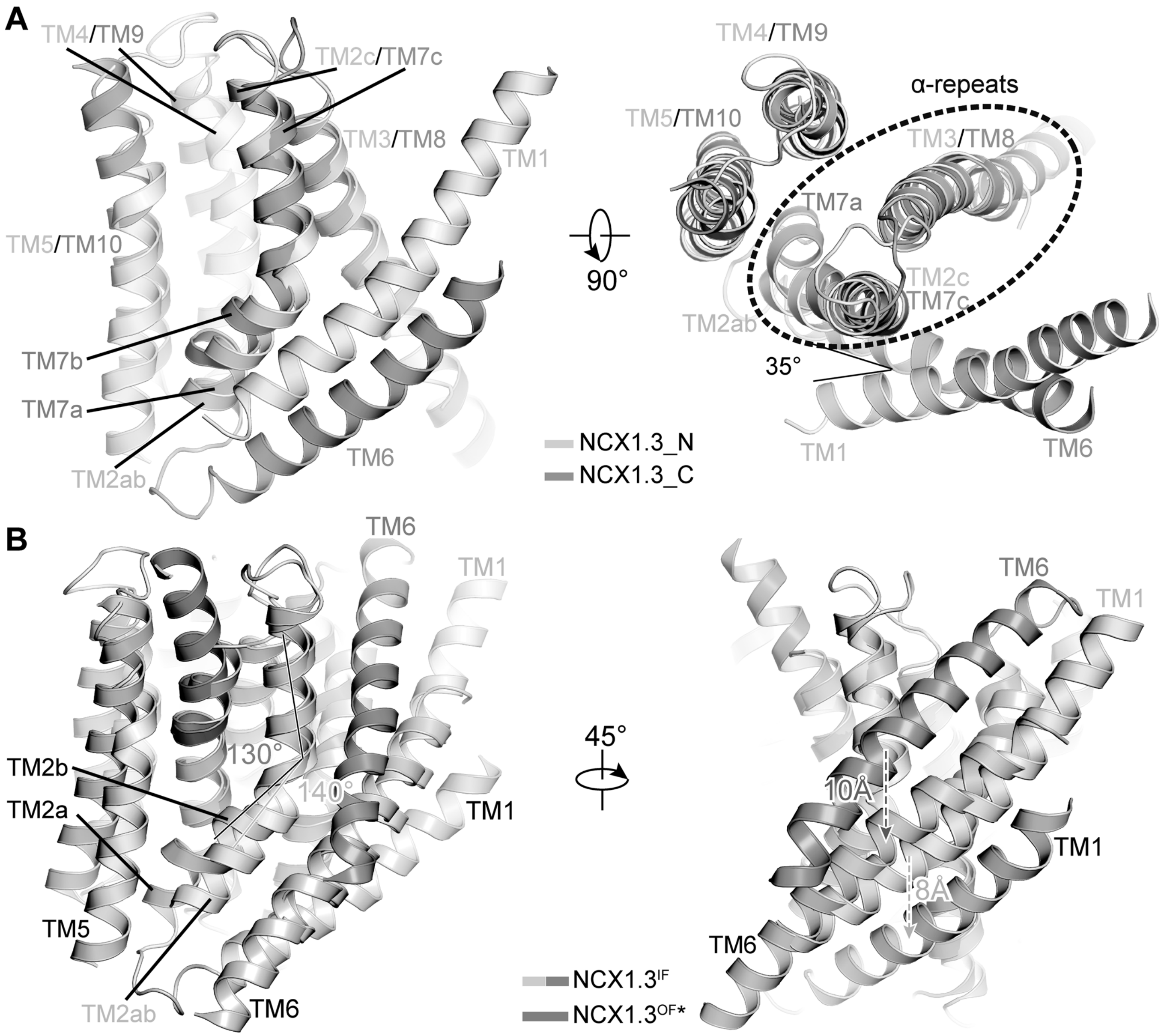

Figure EV2. Structural comparison of NCX1.3 between N-terminal and C-terminal. Related to Fig. 2 and Fig. 4.

(**A**) Structural comparison between NCX1.3_N (N-terminal half of NCX1.3, in pink) and inverted NCX1.3_C (C-terminal half of NCX1.3, in dark blue) viewed from parallel to the membrane and the extracellular side, respectively. The α-repeats are marked as the green dotted oval. The angle between TM1 and TM6 of NCX1.3_N is indicated. (**B**) Structural comparison between NCX1.3 (in pink and dark blue) and outward facing NCX1.3 model (NCX1.3^{OF*}, in gray) model using the core domain (TM2–5 and TM7–10) viewed parallel to the membrane and with rotation of 45°. NCX1.3^{OF*} model is the inverted structure of NCX1.3^{IF} due to the symmetry of NCX1.3 structure and its ion-binding sites. TM1 to TM5 were superposed on TM6 to TM10 in an analogous manner for mjNCX. The helix bending between TM2ab and TM2c of NCX1.3 and NCX1.3^{OF*} are indicated, respectively. The displacements of TM1 and TM6 between NCX1.3 and NCX1.3^{OF*} are indicated.

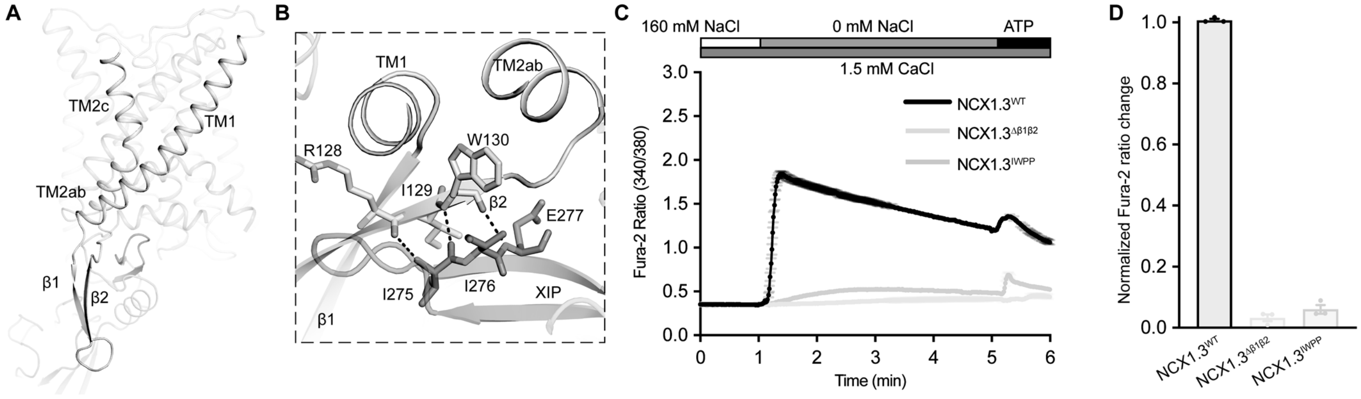

Figure EV3. Structural and functional characterization of β sheets between TM1 and TM2 in NCX1.3. Related to Fig. 3.

(**A**) The position of β1 and β2 in the overall structure. TM1, β1, β2 and TM2 are shown as pink cartoons. (**B**) Detailed binding sites between β2 and XIP region. The side chains of key residues are displayed in sticks and black dashed lines indicate potential hydrogen bonds. (**C**) Representative averaged traces of cytosolic Ca^{2+} measurements for NCX1.3^{WT}, NCX1.3$^{\Delta\beta1\beta2}$ and NCX1.3^{IWPP}. (**D**) Normalized Fura-2 ratio change values are measured as the difference between the initial ratio values and the peak ratio values for NCX1.3^{WT} ($n = 3$), NCX1.3$^{\Delta\beta1\beta2}$ ($n = 3$) and NCX1.3^{IWPP} ($n = 3$).

 © The Author(s)

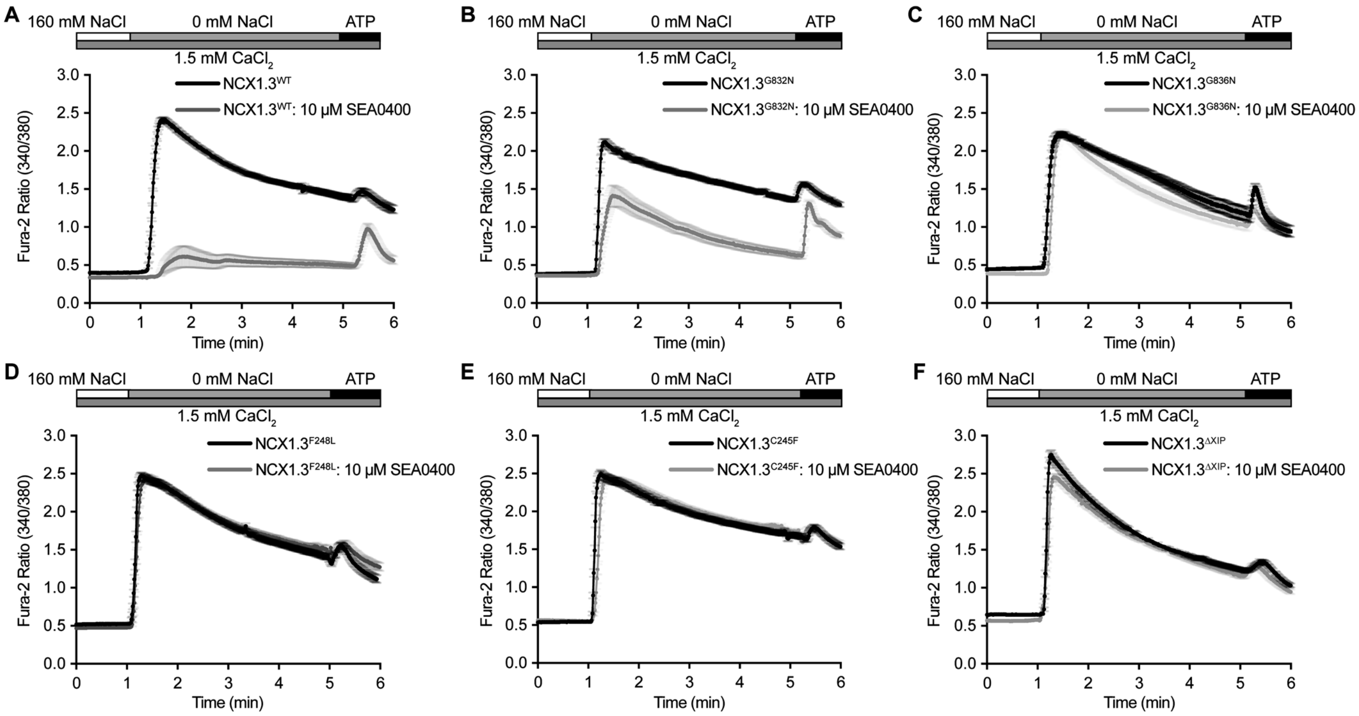

Figure EV4. Effect of SEA0400 on NCX1.3 activity in HEK293 cells. Related to Fig. 1, Fig. 3, and Fig. 4.

(**A–F**) Representative averaged traces of cytosolic Ca^{2+} ($[Ca^{2+}]_i$) measurements showing $[Ca^{2+}]_i$ increase in HEK293 cells of wild-type and mutant variants by calcium imaging experiment. The HEK293T cells are transfected with $NCX1.3^{WT}$ (**A**), $NCX1.3^{G832N}$ (**B**), $NCX1.3^{G836N}$ (**C**), $NCX1.3^{F248L}$ (**D**), $NCX1.3^{C245F}$ (**E**) and $NCX1.3^{\Delta XIP}$ (F). These mutants are designed to disturb SEA0400 binding pocket of NCX1.3. Each panel depicts superimposed traces representing the $[Ca^{2+}]_i$ response when Na^+-free $NMDG^+$ buffer alone, or in the presence of SEA0400 (10 µM). The change of solution was marked by time breaks. The Krebs' buffer including 160 mM NaCl was changed Na^+-free $NMDG^+$ buffer (0 mM NaCl) at one minute. Subsequently, 500 µM ATP was added at the end as a control to measure cell viability. The Fura2 ratio (340/380) was used as a quantitative indicator of intracellular $[Ca^{2+}]$. For each experiment, data shown are representative of at least three experiments with >20 cells for each condition. Error bars are defined as mean ± SEM.

© The Author(s)

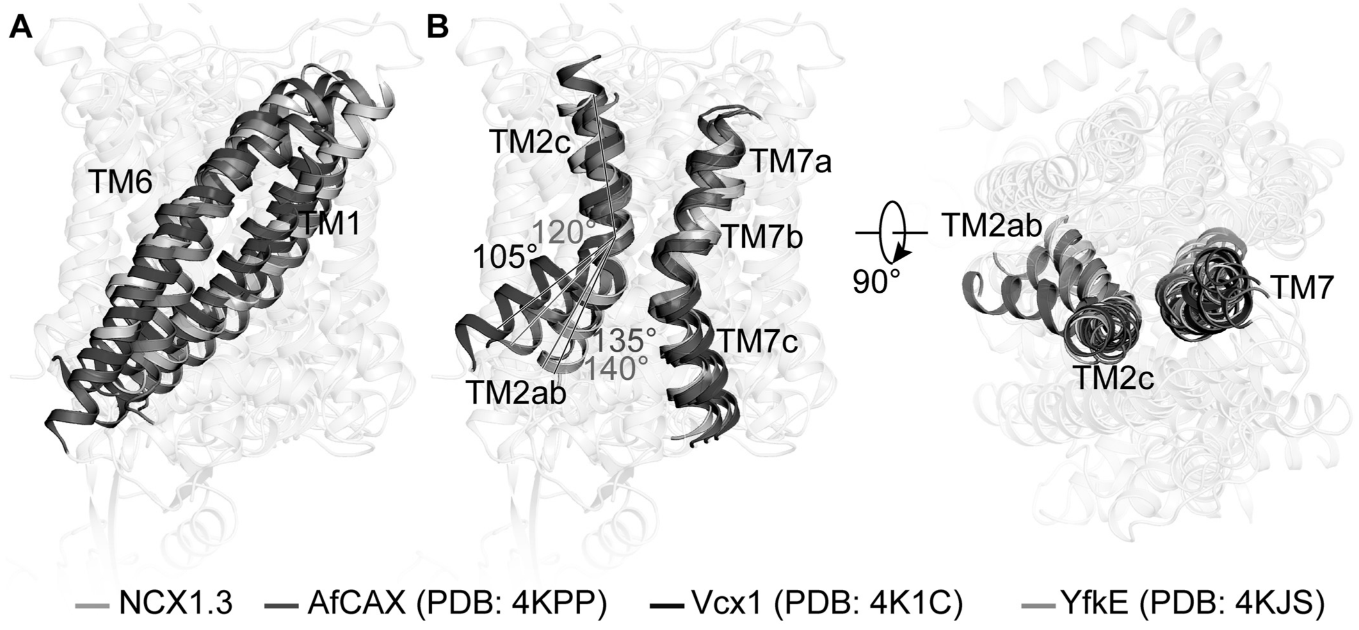

Figure EV5. Superimposition of the core domain (TM2-5 and TM7-10) among NCX1.3, AfCAX, Vcx1, and YfkE. Related to Fig. 4.

(**A**) Structural comparison among NCX1.3 (in gray), AfCAX (in red), Vcx1 (in green) and YfkE (in yellow) using the core domain (TM2–5 and TM7–10). Structural changes of the TM1 and TM6 helices are shown. (**B**) Structural changes of the TM2 and TM7 helices. The angles of helix bending between TM2ab and TM2c among them are indicated.

 © The Author(s)
